# A cyclical wildfire pattern as the outcome of a coupled human natural system

**DOI:** 10.1038/s41598-022-08730-y

**Published:** 2022-03-28

**Authors:** Farshad Farkhondehmaal, Navid Ghaffarzadegan

**Affiliations:** Department of Industrial and Systems Engineering, Virginia Tech, Blacksburg, USA

**Keywords:** Climate sciences, Ecology, Environmental social sciences, Natural hazards

## Abstract

Over the past decades, wildfire has imposed a considerable cost on natural resources and human lives. In many regions, annual wildfire trends show puzzling oscillatory patterns with increasing amplitudes for burned areas over time. This paper aims to examine the potential causes of such patterns by developing and examining a dynamic simulation model that represents interconnected social and natural dynamics in a coupled system. We develop a generic dynamic model and, based on simulation results, postulate that the interconnection between human and natural subsystems is a source of the observed cyclical patterns in wildfires in which risk perception regulates activities that can result in more fire and development of vulnerable properties. Our simulation-based policy analysis points to a non-linear characteristic of the system, which rises due to the interconnections between the human side and the natural side of the system. This has a major policy implication: in contrast to studies that look for the most effective policy to contain wildfires, we show that a long-term solution is not a single action but is a combination of multiple actions that simultaneously target both human and natural sides of the system.

## Introduction

Wildfire is endangering human life, natural resources, forest conservation, and wildlife^1–4^. According to the National Interagency Fire Center, in 2020, more than 52,000 wildfire incidents in the United States burned about 3.64 million hectares^5^. In California alone, it was estimated that about 30 people died due to wildfires during the first 9 months of 2020^6^. In addition, the tragic 2018 Camp Fire incident of Paradise, California, arguably the most destructive and deadliest wildfire in California's history, resulted in at least 85 civilian fatalities and burned over 60,702 hectares, destroying more than 18,000 structures^7^. Furthermore, the problem is not limited to the United States: Wildfire is a global challenge affecting different regions worldwide, with recent catastrophic events in countries such as Australia, Brazil, Greece, Algeria, France, Turkey, and Indonesia. Given the trends, the problem of wildfires and their increasing catastrophic consequences are of immense policy relevance.

**Figure 1:**
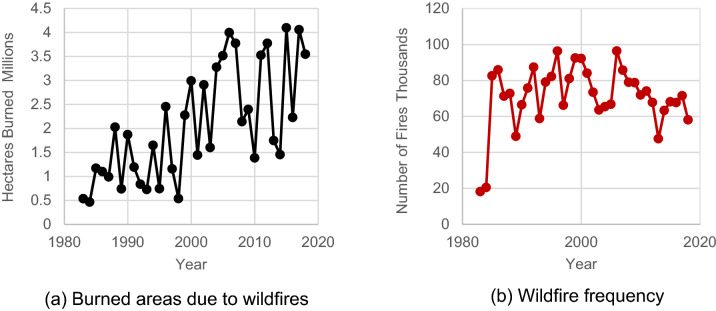
Wildfire in the U.S.1983–2018 (data from www.nifc.gov).

Understanding and predicting the occurrence of wildfires is vital for taking proper policy actions to mitigate the risks and minimize associated consequences^8–11^. An examination of historical trends of wildfires reveals puzzling cyclical patterns in fire incidences, with increasing amplitude for the consequences of fire in many areas around the globe, including the U.S. As Fig. 1 shows, in the U.S., we have experienced an overall increasing trend of the burn rate due to wildfire, with periodic fluctuations. Interestingly, although the overall pattern of the number of fires does not follow the burn rate trend, it does show periodic oscillations. Finding the drivers of such patterns is an area of concern for natural scientists, policy researchers, and policymakers.

Wildfires start with initial fire ignitions, which can be caused by nature through lightning or reckless human behavior. The occurrence of natural fires through lightning depends on weather conditions and shows a seasonal pattern^12^. Human-caused ignition, on the other hand, can also cause large-scale fires. In fact, in the U.S., human-ignited wildfires account for approximately 84% of wildfires nationwide^13^. In addition, factors such as abandoned campfires, arson, and fireworks can lead to human-ignited fires^14,15^. Humans also indirectly contribute to wildfire through activities that worsen climate change^16^. The release of greenhouse gases into the atmosphere, including carbon dioxide and methane, contributes to higher temperatures^17^. A warmer climate leads to drier vegetation in forests and increases the risk of massive wildfires^18^. Furthermore, deforestation for land development reduces the ability of the forest to absorb greenhouse gases, which ultimately causes a further increase in temperature^19,20^.

Despite the importance of direct human and natural contributions to wildfire, the focus of most past modeling studies has been solely on one of these two categories of causation. Touboul and colleagues developed simulation models of dynamic interactions among different kinds of vegetation such as grass and forest trees. They showed that for a wide range of scenarios, the composition of vegetation can oscillate over time^21^. Such models focusing on natural-system dynamics can explain long-term oscillatory patterns that emerge from forest recovery delays after a wildfire. On human contributions, several statistical models have pointed to a correlation between human settlement in the wildland-urban interface (WUI) and fire activity^22–24^. In these models, human-risk perception is often an exogenous factor that affects fire. We understand that both natural and human sides of the problem are important. In fact, it has been argued for a long time that accounting for dynamic connections between social and ecological systems is essential in developing sustainable environmental policies^25^. Therefore, we hypothesize that the interaction between natural and human systems contributes to wildfire dynamics, increasing their complexity and mitigation challenges. To develop proper policies, attention should be paid to both sides of the larger system and the interactions between the two. Our primary objective in this paper is to explore potential causes of such patterns by developing and examining a feedback-rich dynamic simulation model that represents both social and natural dynamics in a coupled system.

**Figure 2:**
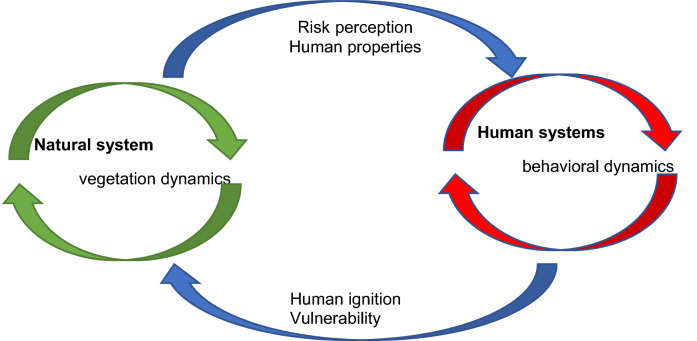
Our study framework of wildfire as an outcome of a coupled human-natural system (adapted from https://www.nsf.gov/pubs/2018/nsf18503/nsf18503.htm and adjusted for the case of wildfires).

Figure 2 presents our study framework, which is in line with a body of the ecological literature that examines a family of phenomena referred to as coupled human-natural systems (This area has been a major area of investigation at the U.S. National Science Foundation). The framework includes dynamics specific to vegetation (natural systems) and human systems (behavioral dynamics). In interaction, the two pieces are connected through the human sector that receives information regarding recent fire cases and influences the human risk perception, as the perceived information influences the fire risk^26^. Humans contribute to fire through human-caused ignition or the development of vulnerable properties based on their risk perception.

## Background: models of disasters

While our focus is on the specific problem of wildfire, it is important to pause and offer a quick review of various modeling approaches of similar natural disasters, mainly from a methodological standpoint. There is a wide range of modeling approaches applied to natural-disaster studies in general and wildfires in particular. Such modeling can be differentiated based on their unit of analyses, time frames, mathematical modeling techniques, boundaries, and specific application cases.

A large body of natural disaster models has been devoted to spatial modeling^27–29^. In a typical spatial wildfire model, the goal is to replicate fire progression throughout different regions. Such models are powerful in showing how, in what sequence, and the timing of different areas may become fire susceptible. Spatial models can also take different forms depending on the geographical units of analysis (e.g., state, county). Connection networks between different units can affect fire progress, and such models become more useful as they move toward modeling network structures.

The second group of models of natural disasters includes agent-based individual-level models. Models of evacuation often take such levels of analysis and explore the flows of individuals after a disaster^30,31^. In the wildfire context, with a focus on fire progression, agent-based models may consider vegetation units as agents. Such models would lead to a spatial model of interacting elements that depict endogenous fire propagation from one unit of vegetation to another. Rahmandad and Sterman^32^ stressed that in many contexts, detailed agent-based models may not go beyond what one could learn from an aggregate differential-equation model, especially when the heterogeneities across the agents are limited and connection networks are symmetrical and almost complete.

On the other side, aggregate natural disaster models exist in which vegetation is often modeled with a few major variables but no regional details^33,34^. As compartmental models, these often include differential equations and formulate vegetation flows and aging of trees in a dynamic framework^35^. Within aggregate models, the extent to which variables are treated as endogenous variables (that is, they respond to changes in state variables) is a significant factor for differentiating. Simon Levin and colleagues^21,36,37^ offered different variations of aggregate, differential-equation models of vegetation. An interesting outcome of such models from a complex-systems point of view is the depiction of bifurcation that the model's outcomes substantially change from a steady-state to a goal-seeking or s-shaped behavior or even long-term oscillations for different ranges of parameter values.

Within the system dynamics community, there is also a rich body of literature on modeling environmental problems^25,38,39^. Deegan^40^ has conducted methodologically relevant work in a slightly different natural-disaster setting. He modeled flood-damage dynamics in a typical flood-prone community, considering long-term community reactions to recent floods and related damages. Deegan focused on hypothetical flood cases, intending to show how seemingly similar external events (here, major rain) can cause different damage levels depending on the community's reactions and investment in vulnerable properties. What differentiates his work from others is that Deegan's model is feedback-rich, and dynamic outcomes are created within the model rather than by an external time series^41^. In some respects, our approach to modeling wildfires resonates with Deegan's flood mitigation work by looking at vulnerability as an endogenous property of the system affected by human risk perception.

What makes these aggregate models powerful is that they are relatively small (have fewer equations), and when the details are removed, they turn the focus on system responses and feedback loops without losing many systems-level insights^32^. Modelers can also better communicate insights from small models with stakeholders^42^. It is important to note that small, powerful models are not easy to build, and they are often the result of many rounds of complex and detailed modeling^43^, which has also been the case in our study. Given our problem scope, we follow the same modeling approach.

## Model structure and key formulations

**Figure 3:**
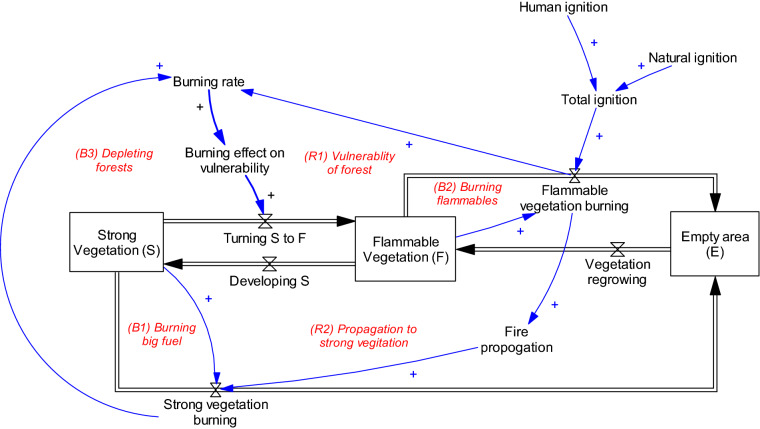
A stock-flow diagram of vegetation. Note: Sock variables represent the state of the system and are shown in boxes (strong vegetation, flammable vegetation, and empty area), and flows, representing change in state variables are depicted by valve signs. Causal influences are shown with blue links, where a plus sign in X →  + Y indicates that X and Y move in the same direction (${\text{X}} \to + {\text{Y}} \Leftrightarrow {{\partial {\text{Y}}} \mathord{\left/ {\vphantom {{\partial {\text{Y}}} {\partial {\text{X}}}}} \right. \kern-\nulldelimiterspace} {\partial {\text{X}}}} > 0$). A minus sign on a causal arrow indicates that the variables move in the opposite direction (${\text{X}} \to - {\text{Y}} \Leftrightarrow {{\partial {\text{Y}}} \mathord{\left/ {\vphantom {{\partial {\text{Y}}} {\partial {\text{X}}}}} \right. \kern-\nulldelimiterspace} {\partial {\text{X}}}} < 0$). For detailed information on causal loop diagrams, see Sterman^44^.

Different models use varying terms to represent vegetation heterogeneities in a forest area. For the purposes of parsimony, our model represents the entire forest area by two simple stock variables of areas occupied by strong vegetation (*S*) and occupied by flammable vegetation (*F*) all of which are shown as stock variables in Fig. 3 (variables in boxes). Strong vegetation is often resistant to fire, and only large-scale fires can burn them. Highly flammable vegetation includes damaged or any vegetation that can burn fast (including grass). This type of vegetation can burn quickly, and lightning or human ignition often affects flammable vegetation first. Burning can cause fire propagation to strong vegetation. While our figure is a simple representation of forest areas, the logic is consistent with studies that have offered more detail on vegetation types.

In this model, loops B1 and B2 represent the deterioration of strong and flammable vegetation through fire. As stated, fire can increase the vulnerability of strong vegetation by burning the surrounding area and making it more susceptible to fire. This mechanism is shown by loops R1 (burning of flammable vegetation further increases flammable vegetation) and B3 (burning of strong vegetation makes other strong vegetation vulnerable to fire). In this study we assume both areas occupied by strong and flammable vegetation are homogenous. By taking this simplistic assumption, we believe that the model’s behavior is independent from spatial details of the vegetations. This assumption, which is called universality, have been previously considered in different compartmental modeling studies^37^.

We base our model for the unit of forest area, which leads to the fact that the empty area (E) of the forest can be determined by the following equation:

1 $$E = 1 - F - S$$

We then can represent the relation between the stock variables by the following differential equations.

2 $$\frac{dS}{{dt}} = \frac{F}{{\tau_{1} }} - \left( {\alpha + \gamma_{S} } \right)S$$

3 $$\frac{dF}{{dt}} = \frac{E}{{\tau_{2} }} + \alpha S - \left( {\frac{1}{{\tau_{1} }} + \gamma_{F} } \right)F$$

where $\gamma_{S}$ and $\gamma_{F}$ are the fractional burning rate of strong and flammable vegetation, respectively; $\alpha$ is the rate of making strong vegetation to flammable; $\tau_{1}$ is the average time for flammable vegetation to become strong; and $\tau_{2}$ is the average time for the empty space to grow flammable vegetation, where often $\tau_{2} < < \tau_{1}$. Thus, the total burn rate from both types of vegetation (*B*) is

4 $$B = \gamma_{F} F + \gamma_{S} S.$$

In this equation, $\gamma_{F}$, the rate at which flammable vegetation is burned, is a function of the total of human and natural ignitions. However, $\gamma_{S}$, the fractional burning rate of strong vegetation depends on the burning rate of flammable vegetation and happens when fire propagates in the forest—i.e., $\gamma_{S} = f\left( {\gamma_{F} F} \right)$. We formulate *f* using a sigmoid function (Table 1). Furthermore, $\alpha$, the rate of becoming flammable for strong vegetation as a result of fire is $\alpha = \sigma B$ where $\sigma$ is the burning effect on vulnerability.

**Table 1:** Parameter values for a base run simulation.

Parameter	Value	Unit
*τ*_1_	2	Year
*τ*_2_	10	Year
$\delta_{1}$	0.5	Year
*S*	Initial value: 0.5	Million hectares
*F*	Initial value: 0.4	Million hectares
$\gamma_{S}$	$0.8*\left( {1 + e^{{ - 5*\left( {\frac{{\gamma_{F} F}}{n} - 1} \right)}} } \right)^{ - 1}$	1/year
n	0.1	Million hectares
*I*_*N*_	0.5	Scalar
*I*_*H*_	Initial value: 0.3	Scalar
*V*	Initial value: 0.4	Million hectares
$\sigma$	0.05	1/Million hectares

**Figure 4:**
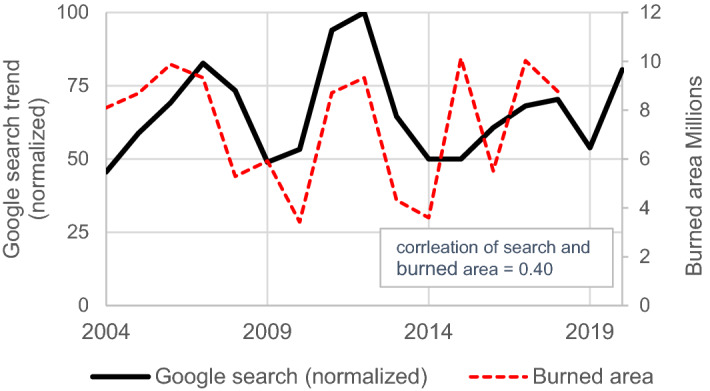
Google search trend for "wildfire" in the U.S. and its correlation with the annual burned area.

Generally, the public attitude towards making risky decisions is influenced by their level of risk perception. In the case of wildfire, there is a wide range of evidence that people's attention to the problem and possibly the associated risk perception has changed over time. Figure 4 depicts the frequency of Google searches for the word "wildfire" in the U.S. The trends are oscillatory, and there is a 0.4 correlation between search and area burned from 2004 to 2018.

There is a body of research focused on how the perception of wildfire risk is associated with mitigation actions^45–48^. A study of a fire-prone area in Colorado revealed that a single extreme wildfire does not significantly impact risk perception^47^. Furthermore, evidence on people's fire-risk perception shows that any change in fire risk perception does not last more than a couple of years^48^. We construct the effect of risk perception on human actions based on the abovementioned research with two important characteristics. First, the overall wildfire activity in recent years shapes people's fire risk perception; second, the effect of wildfire on people's perception vanishes as time pass.

**Figure 5:**
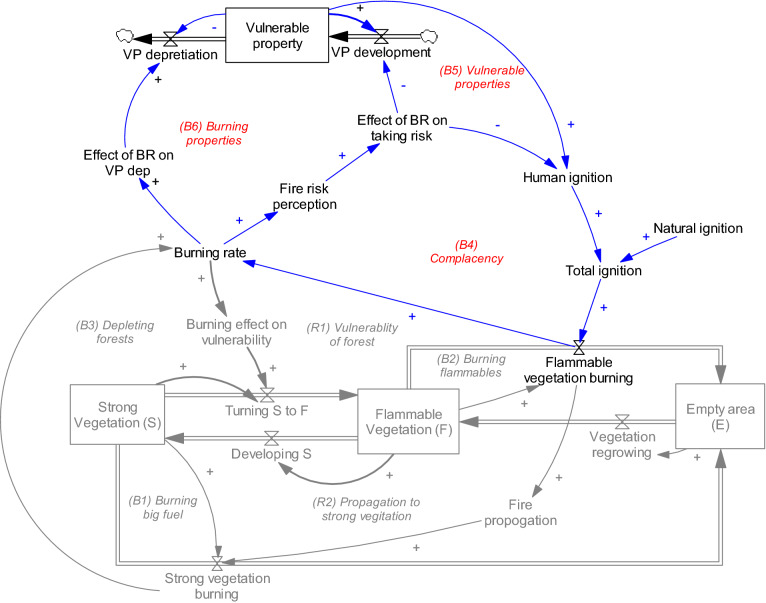
The human subsystem as connected to the natural subsystem (grey).

We include two major mechanisms to depict the effects of change in risk perception, as shown in Fig. 5. The loop B4, complacency, represents the human contribution to fire through reckless behaviors, which can cause fire ignition. Loop B5, vulnerable properties, represents property building in forest areas. Such properties increase human interaction with the natural environment and the likelihood of human-made ignition. We also consider the fact that such properties might be targets of fire themselves, loop B6.

In this model risk perception, $\overline{B}$ is formulated as a $\delta_{1}$-year lagged variable of burn rate ($B$), assuming there is no systematic bias in risk perception. Total ignition of *I* includes human-caused ignition ($I_{H}$) and natural ignition due to lightening ($I_{N}$), with the latter, assumed as constant in our model. Several studies in different regions of the world (Spain, Canada, and the United States) suggest Human-caused ignition increases by human settlements in the area^49–51^. We also consider the number of human ignitions is inversely related to their risk perception. Assuming human settlements are represented by vulnerable properties, *V*, we formulate $I_{H}$ as $I_{H} \left( {V,\overline{B}} \right)$ where $\frac{{\partial I_{H} }}{\partial V} > 0$ and $\frac{{\partial I_{H} }}{{\partial \overline{B}}} < 0$. For the purposes of parsimony, we formulate effect of $\overline{B}$ on $I_{H}$ using a linear function (see the Appendix).

Although Martin et al. ^43^ discussed how different stakeholders (including insurance companies and federal agencies) could increase the sensitivity of humans to risk perception, they did not provide any quantitative estimation of this value.

Finally, vulnerable properties, *V*, which can change over time is formulated as

5 $$\frac{dV}{{dt}} = V\left( {\theta E_{bt} - \rho } \right)$$

The term, $\theta E_{bt}$ represents property development and is assumed to be proportional to the current properties and negatively affected by risk perception. The inverse relationship between perceived risk and vulnerable property expansion is a proxy for external conditions like zoning since building policy decisions is too complex to be modeled directly as they are very context-specific and involve political decisions^52^. While some studies suggest no relationship between natural disaster occurrence and community development programs, others consider economic intensive (such as insurance policy) to cause reduction in development pace as disasters increase^53,54^. Here we acknowledge that there is no general agreement on the effect of natural disaster and development programs and build the model for areas where such a relationship is proved to exist. The term $\rho V$ represents the demolition of properties. Demolition in our model is mainly due to the fire, that is, $\rho = \rho \left( B \right)$.

## Parameter values

The introduced model is generic and can be simulated for a wide range of parameter values. Table 1 reports parameter values used for base run simulations. Some of the values are consistent with the literature, while others are selected to examine variation of conditions in different forestry settings. To make sure the modeling result is robust, we perform sensitivity analysis for a wide range of variables. The sensitivity result suggests the model outcome is consistent with the base run (See Appendix 2).

Our simulation experiments include a base run simulation and a range of policy and scenario tests as listed in Table 2. The table also provides details on how each test is implemented in our analysis. Specifically, we analyze the linkage between natural dynamics and human perception and its consequences on fire development by changing the sensitivity of risk perception to the burn rate (Test T2). We then examine the effects of four different policies: limiting the development of vulnerable properties (P1), prescribed and controlled burning of flammable vegetation (P2), effective firefighting that limits penetration of fire from flammable vegetation to strong vegetation (P3), and clear cutting (P4) which remove part of strong vegetation trees and change it to the empty area^55^.

**Table 2:** Simulation experiments.

Simulation tests	Operationalization
T1: Base run	Parameter values in Table 1 are used for this scenario
T2: Coupling effect	Sensitivity of risk perception to burn rate is changed by changing risk perception delay from 1 year (base run) to
	0.5 year (T2a: higher sensitivity),
	2 years (T2b: lower sensitivity), and
	100 years (T2c: least sensitivity—almost disjointed systems)
T3: Policy tests	Three policies are tested
	P1: Limit vulnerable property development
	P2: Prescribed burning
	P3: Effective firefighting
	P4: Clear cutting
	P1 is implemented by making vulnerable property development equal to 1%. P2 is implemented by adding outflow from flammable vegetation to an empty area with the value of $\omega F$, where $\omega$ is the percent of prescribed burning set at 0.2/year. P3 is implemented by changing $\gamma_{S}$ to 10% of its current value. Finally, P4 implemented by adding outflow from strong vegetation to the flammable vegetation with the value of $\vartheta S$ where $\vartheta$ is the percent of clear cutting set to 0.2/year (see Appendix 3 for detail)

## Simulation results

### Base run simulation

**Figure 6:**
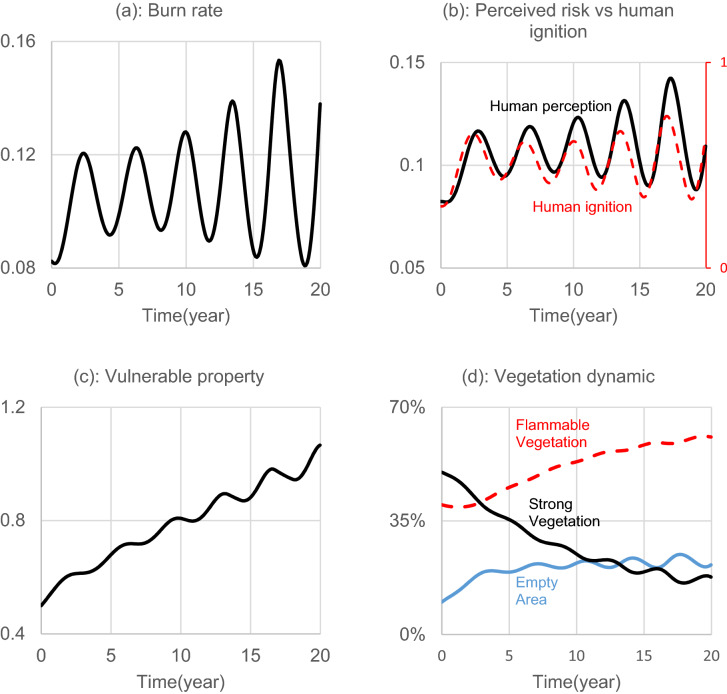
Base run simulation for a 20-year run of the model.

Figure 6 shows the results of the base run simulation. In this scenario, strong vegetation declines over time, while the empty area and flammable vegetation have increasing trends. As such, more fuel would be available for burning, and the wildfire can burn broader areas. Panel (a) shows an oscillatory trend for the burn rate with an average upward trend (To make sure the oscillatory behavior of the model does not fade, Appendix 4 shows the simulation result for 100 years). The observed pattern in the burn rate can be traced back to the patterns of human ignition (Panel b), and the growing trend of vulnerable properties (Panel c). In addition, the results show the long-term declining trend of strong vegetation in our base line simulation (Panel d); over time, stronger vegetation is replaced by flammable vegetation which can lead to more fire. This change in vegetation composition effectively increases the average burn rate. Over time, with more flammable vegetation and with the expansion of vulnerable properties, the likelihood of human-made ignition increases.

### Coupling effects

**Figure 7:**
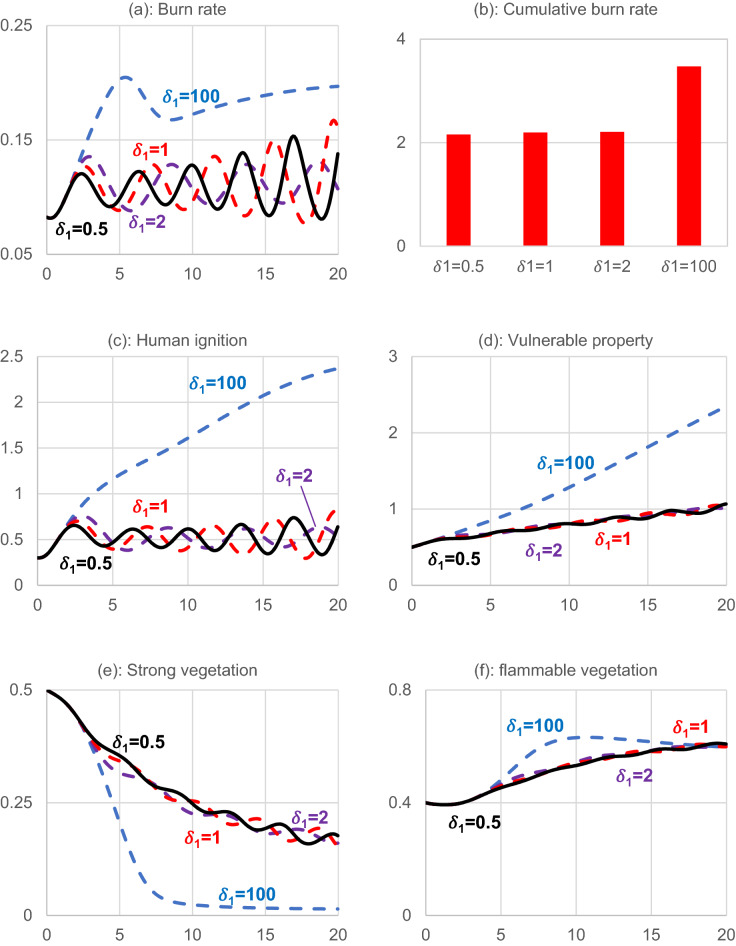
Coupling effect analysis for 20 years. Human ignition unit is Ignition/year, and vulnerable property unit is a million hectares. Strong vegetation and flammable vegetation are provided as the ratio that each occupied the forest area.

Figure 7 shows how the relation between perceived fire risk and the burn rate influences the system. The black line is the base run simulation for comparison. The blue dashed line depicts the condition in which risk perception changes extremely slowly, and the human system is almost disconnected from the natural system. In this situation, if humans underestimate the fire potential, the system burns down nature, resulting in a catastrophic environmental outcome as depicted in panel (a). Panel (a) shows that the burn rate overshoots in the short term but relatively declines due to less remaining natural resources to burn.

Panel (b) displays the total burn rate throughout the study time to cast further insight into the burn rate sensitivity to perceived risk. The overall burn rate does not significantly change when the risk perception changes from 0.5 to 2, indicating the difference among burn rates in panel (a) is more about the fluctuation timing, but not the size. However, an additional rise in the sense of risk greatly raises the overall burn rate, as seen in panel (a).

In the case of prolonged change in risk perception, human ignition continues to increase (panel c) as the perceived risk changes slowly. Furthermore, vulnerable properties are being built faster than their demolition (panel d). A slighter delay in perception leads to a higher frequency of oscillation as depicted in the graphs by the red dashed lines and a longer delay in a lower frequency oscillation, as shown by the purple graphs. Overall, the results are not much different from the base run. We are losing forests (panel e) and have periodic burn rates of increasing magnitude over time.

### Policy experiments

**Figure 8:**
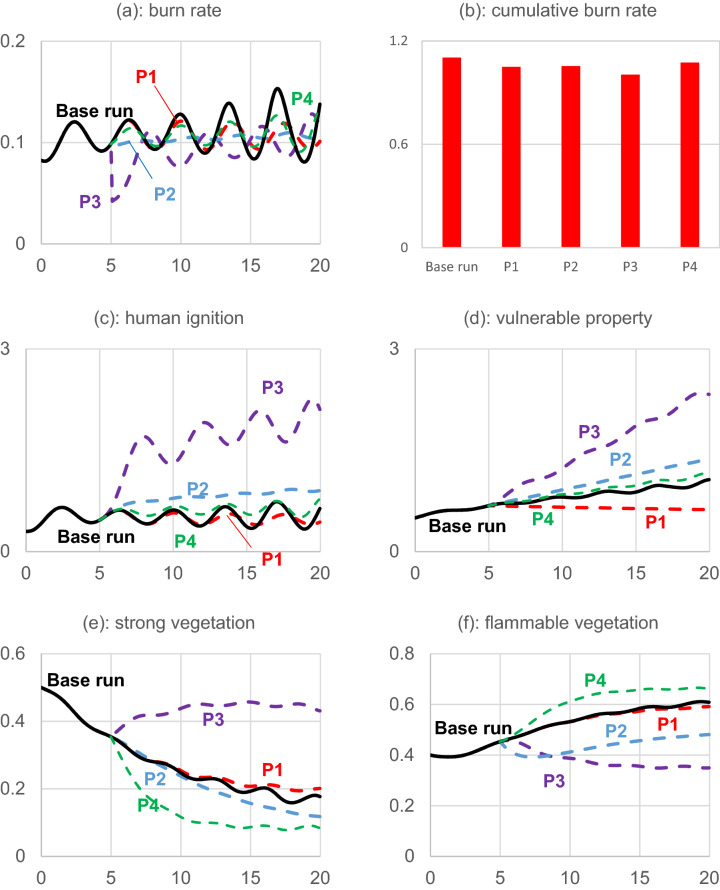
Policy implementation. Note: P1: limits vulnerable property development; P2: prescribed burning; P3: effective firefighting; and P4: Clear cutting. Human ignition unit is Ignition/year, and vulnerable property unit is a million hectares. Strong vegetation and flammable vegetation are provided as the ratio that each occupied the forest area.

Here we examine the impact of implementing four proposed policies introduced in Table 2. To prevent the initial condition and transition periods affecting our comparison of proposed policies, we imposed each policy at the fifth year and compared the total burn rates between 10 and 20 years. Figure 8 shows the effect of these policies on different variables.

Panels (a) and (b) show the burn rate over time and cumulative, respectively. All four policies reduce the burn-rate magnitude compared to the base run. P3 is more effective in early burning-rate reduction compared to other policies, but they ultimately result in similar behavior. It is worth noticing that P1 has the most effect on long-run fluctuation reduction, although its total effect in the time span is less than P3. It seems that firefighting is more effective in the short run, but it fails to dampen the fluctuation and instead limits its growth. This is partly because of the increase in human ignition and settlement due to the success of firefighting in the short run. As a result, people perceive less fire danger and continue to engage in high-risk activities and expand housing in the WUI. The result is further fluctuation in the burn rate even when P3 is implemented. On the other hand, the WUI expansion limitation policy can effectively reduce the burn-rate fluctuation in a timely manner. Implementing P4 causes a reduction in strong vegetation, which leads to flammable vegetation increase. As flammable vegetation is the main fuel for wildfire, this policy cause increase in fuel availability and an increase in the burning rate.

Change in human ignition is provided in panel (c). Different levels of human-made ignition are observable, and the reason is that people adjust their high-risk behavior with burn rate, and not with the number of fires. In the firefighting policy, as for a given level of ignition, the burn rate declines, we observe more risky behavior and more human-made ignition. It is interesting to note that, as panel (c) shows, we end up with more WUI under policies 2, 3, and 4. In fact, the reason is that the firefighting, prescribed burning and clear cutting only affect natural sector of the model, decrease burn rate, which decreases risk perception and in turn result in more WUI development. On the other hand, P1 directly targets WUIs.

Panel (e) displays the change in strong vegetation, which shows that P4 causes the most reduction in forest tree cover as it directly removes strong vegetation. P2 also causes a decrease in strong vegetation compared to the base run. The reason is that burning flammable vegetation damages young trees and prevents them from developing into solid vegetation. On the other hand, P3 has the least effect on strong vegetation by slowing the damage to young trees and confining the fire. Panel (f) shows the flammable vegetation dynamic after imposing each policy. P3 and P2 reduce flammable vegetation more than P1. However, there is an important difference in how these policies cause the reduction in flammable vegetation. In comparing panels (a) and (b), we see that while P3 causes further increases in the strong vegetation, P2 causes an increase in the empty area. P4 is the only policy that increases flammable vegetation by removing the strong vegetation and providing an empty area to be filled with young vegetation.

Overall, it looks like each policy has some marginal effect on containing wildfire, though the magnitudes of effect are not considerable.

### Replication of United States data

**Figure 9:**
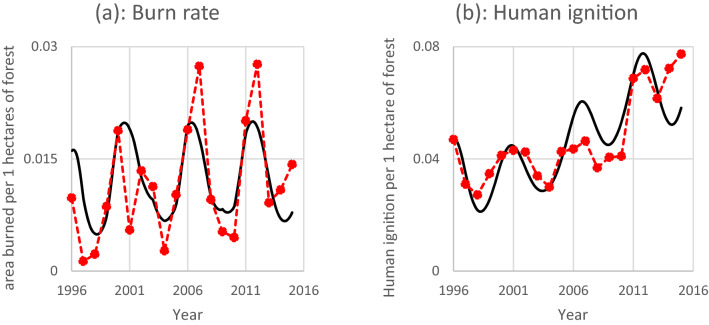
Burning rate and human ignition per unit of forest area. The black line represents the model result, and the red dotted line represents the historical wildfire activity in the conterminous United States.

For model validation, we investigate its ability to fit a single case, United States' wildfires from 1996 to 2015. We utilize the United States Department of Agriculture's wildfire database for the conterminous United States (Short, 2017). The results are shown in Fig. 9. In this figure, simulation of burning rate and human ignition (continuous lines, in black) closely follows the real-world data (dotted lines, in red), and the model fairly replicates the historical trends.

### Combination policy implementation analysis

To better understand the impacts of our policies, we run different pairs of policies simultaneously. The results illustrate the nonlinear incremental impacts between policies. Simply put, it appears that the impact of several policies is enforced when combined synergistically. In other words, applying several policies might have a greater overall impact than the sum of the policies' individual effects and suggests that policymakers should avoid searching for a panacea and adopt a broad range of approaches thoughtfully.

**Figure 10:**
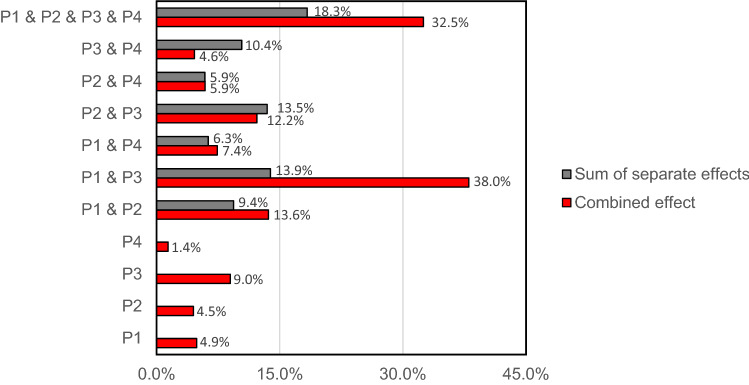
The nonlinear effect of policies. The benefits of implementing multiple policies differ from the sum of the effect of policies. The figure shows the percent of burn rate reduction. Note: P1: limit vulnerable property development; P2: prescribed burning; P3: effective firefighting; and P4: Clear cutting.

The results of multiple policy implementations along with single ones are presented in Fig. 10. For example, P1 and P2 each reduce the total burn rate by 4.9% and 4.5%, respectively. While the summation of these effects is 9.4%, simultaneously implementing P1 and P2 lead to a 13.6% burn-rate reduction—P1 controls the human ignition, and P2 reduces the flammable vegetation stock—together, the burn rate is more affected than if implemented separately. The case is more interesting when P1 and P3 are imposed together. The result is a 38% burn-rate reduction compared to 13.9%, which is the sum of solely implementing each policy. The synergic effect happens because P3 lets the flammable vegetation (mainly young trees) age and become strong vegetation. Furthermore, the P1 also prevents human ignition from growing as fast as a single P3 implementation.

An interesting case happens when P2 and P3 are implemented together. The synergic effect is less than the sum of separate implementation, mainly because both policies affect the vegetation dynamic and not the human factor in the wildfire. P2 and P3 both cause a lower initial burn rate, but due to the reduction in perceived risk of wildfire and expansion of WUI, this effect quickly disappears. This is another evidence for the importance of considering the problem as an interconnected natural and human system, where effective policies should address both sides.

Finally, an interesting result emerges when all policies impose together. Surprisingly, imposing all policies together does not have the most impact on the total burn rate (32.5%), which is less than the P1 and P3 effect (38.0%). The reason relates mainly to the fact P2 and P4 both cause increase in flammable vegetation after empty area filled, which lead to more burning rate after a delay.

### Sensitivity analysis

We conducted a series of sensitivity analysis to check the model's robustness to our assumptions. Specifically, we conducted a Monte-Carlo analysis and changed several parameter values to determine the range of outcomes. The results are reported in Appendix 2. In summary, the focus was on parameters that can take on substantially different values from those assumed in the model, including parameters used for risk perception formulation, its effect on human behavior, such as time to perceive risk and time to change behavior, in addition to fractional burning rate per ignition, average s burning, initial flammable vegetation, initial strong vegetation, human ignition multiplier, and initial vulnerable property. As described in the Appendix, for most of these variables, we changed the corresponding variable up to double its base run value. Moreover, we test different values for initial strong vegetation and initial flammable vegetation changing them between zero and their base run values. Each sensitivity test is the outcome of 2000 simulation runs using a uniformly distributed random distribution of the parameters within the specified intervals. The results are qualitatively robust, and their variability is within reasonable limits (See Figure A1).

## Discussions and conclusion

Wildfire remains one of the major global challenges affecting different regions around the world in all continents. While countries are implementing different policy actions to ameliorate catastrophic outcomes of wildfires, it appears that (a) we are far from addressing this issue on a global scale, and (b) overall, the trends are in the wrong direction, pointing to an increasing magnitude of fires and burned areas. This paper is a response to this challenge. We developed a system dynamics model of wildfire spread in a hypothetical scenario and simulated the effects of several important mechanisms in determining the burn rate, fire frequency, and public risk perception of wildfire. The model included two major sectors of the natural and human subsystem that were connected through the human contribution to ignition and the human risk perception of fire. We simulated the model for a wide range of scenarios that represent different levels of human sensitivity to evolving fires and a range of policy containment measures. Our results show how humans and vegetation determine wildfire activity, defining wildfire as a human-natural coupled system. The findings are important in their relative changes, not their absolute values, because of the model's hypothetical assumption.

We conducted several simulation experiments with the model. The results show a wide range of oscillatory patterns in different scenarios and policy conditions. The base run depicted the possibility of an oscillatory outcome in human-caused ignition and an oscillatory pattern in the burn rate with an overall increasing trend. The decrease in strong vegetation and the increase in vulnerable properties cause an increasing trend in burn rate while dynamics of human perception affect the oscillatory pattern.

Our study contributes to the literature of modeling natural disasters and specifically wildfire studies. We offer the first model of the coupled human-natural system of wildfire. Our study builds on several past models of ecological dynamics^21^, particularly in wildfire dynamics^37^, and extends them to include human interaction with natural systems. The model is generic with the objective of providing insights into human-nature intendencies as related to the problem of wildfire. Our work is different from past spatial models of wildfires. In spatial modeling of wildfires, the human effect is spatially static. Here we show that the same population could ignite a different number of fires and affect the wildfire behavior. Our different approach from past studies results in different outcomes as well. For example, we point to the sources of policy resistance in containing wildfire in terms of how risks are perceived and how properties are built adjacent to natural resources.

Our study resonates with some of the past system dynamics models of other natural disasters^36,37^. We take an endogenous approach to the concept of system vulnerability by considering the human element as a part of the system which both reacts to the problem and contributes to problem. The importance of feedback-rich modeling has previously shown its value in sustainable environmental management, including water quality, waste management, and water supply^25^. Here we propose a similar approach for wildfire management and aim to understand important mechanisms shaping wildfire behavior.

The study has several policy implications. We compared four policies: prescribed burning, vulnerable property control, firefighting effectiveness enhancement and clear cutting. We showed that firefighting effectiveness is more effective in reducing the total burn rate than other proposed policies. More importantly, we showed that simultaneously implementing policies can lead to a synergic effect that can surpass the sum of the effect of solely implementing the same policies. For example, while controlling development of vulnerable properties and effective firefighting each reduce the burn rate 4.9% and 9%, respectively, performing both policies results in a 38% burn rate reduction. Such a synergic effect points to the absence of a silver bullet in controlling wildfires, suggesting that effective policies should target both human- and natural-sectors of the system to maximize their effectiveness. In other words, since wildfire is an outcome of a coupled system that includes highly interdependent human and nature sectors, one cannot solve it by solely focusing on one sector.

This study has several limitations which lead to future avenues for further explorations. We purposefully kept the model simple with a focus on the interdependencies between the human and natural sectors of the model. For example, a detailed examination of spatial dynamics in this context, which will require a larger scale model, will be potentially insightful and have policy implications. With a spatial model, policy analysis can be expanded to include effects of a wide range of silvicultural policies (such as thinning), and with more detailed behavioral models that capture human heterogeneities one may offer behavioral policy insights. A full calibration of the model on a global scale and inclusion of fire penetration across different regions are other potential avenues of expansion.

## Supplementary Information

Supplementary Information.

## References

[1] Scott, J. H., Thompson, M. P. & Calkin, D. E. A wildfire risk assessment framework for land and resource management (2013). 10.1002/ieam.1365 22987567

[2] McFarlane BL, McGee TK, Faulkner H (2011). Complexity of homeowner wildfire risk mitigation: An integration of hazard theories. Int. J. Wildland Fire.

[3] Radeloff VC (2018). Rapid growth of the US wildland-urban interface raises wildfire risk. Proc. Natl. Acad. Sci..

[4] Fowler M (2019). A dataset on human perception of and response to wildfire smoke. Sci. Data.

[5] Philantropy, C. F. D. 2020 North American Wildfire Season (2020).

[6] NBCNews. *Deadly Fires in California Have Claimed at Least 30 Lives This Year.* [30 September 2020]. https://www.nbcnews.com/news/us-news/deadly-fires-california-have-claimed-least-30-lives-year-n1241632 (2020).

[7] Post, W. The deadliest, most destructive wildfire in California’s history has finally been contained (2018).

[8] Catry FX, Rego FC, Bação FL, Moreira F (2010). Modeling and mapping wildfire ignition risk in Portugal. Int. J. Wildland Fire.

[9] De Vasconcelos MP, Silva S, Tome M, Alvim M, Pereira JC (2001). Spatial prediction of fire ignition probabilities: Comparing logistic regression and neural networks. Photogramm. Eng. Remote Sens..

[10] Johnson EA, Miyanishi K (2001). Forest Fires. Behaviour and Ecological Effects.

[11] Bonazountas M, Kallidromitou D, Kassomenos P, Passas N (2005). Forest fire risk analysis. Hum. Ecol. Risk Assess..

[12] Loeb LB (1949). The mechanism of lightning. Sci. Am..

[13] Balch JK (2017). Human-started wildfires expand the fire niche across the United States. Proc. Natl. Acad. Sci..

[14] Prestemon JP, Butry DT (2008). The Economics of Forest Disturbances.

[15] Bartlein, P. J., Hostetler, S., Shafer, S., Holman, J. & Solomon, A. In *5th Symposium on Fire and Forest Meteorology.* 16–20.

[16] Abatzoglou JT, Williams AP (2016). Impact of anthropogenic climate change on wildfire across western US forests. Proc. Natl. Acad. Sci..

[17] Fleming JR (1999). Joseph Fourier, the ‘greenhouse effect’, and the quest for a universal theory of terrestrial temperatures. Endeavour.

[18] Westerling AL, Hidalgo HG, Cayan DR, Swetnam TW (2006). Warming and earlier spring increase western US forest wildfire activity. Science.

[19] Fiddaman, T., Sterman, J., Jones, A. P., Sawin, E. R. & Siegel, L. S. C-ROADS: Climate-rapid overview and decision-support simulator (2008).

[20] Rooney-Varga JN (2020). The climate action simulation. Simul. Gaming.

[21] Touboul JD, Staver AC, Levin SA (2018). On the complex dynamics of savanna landscapes. Proc. Natl. Acad. Sci..

[22] Pew K, Larsen C (2001). GIS analysis of spatial and temporal patterns of human-caused wildfires in the temperate rain forest of Vancouver Island, Canada. For. Ecol. Manag..

[23] Matin MA (2017). Understanding forest fire patterns and risk in Nepal using remote sensing, geographic information system and historical fire data. Int. J. Wildland Fire.

[24] Vilar L, Woolford DG, Martell DL, Martín MP (2010). A model for predicting human-caused wildfire occurrence in the region of Madrid, Spain. Int. J. Wildland Fire.

[25] Stave K (2010). Participatory system dynamics modeling for sustainable environmental management: Observations from four cases. Sustainability.

[26] Langarudi SP, Silva CG, Fernald AG (2021). Measure more or report faster? Effect of information perception on management of commons. Syst. Dyn. Rev..

[27] Keane RE, Drury SA, Karau EC, Hessburg PF, Reynolds KM (2010). A method for mapping fire hazard and risk across multiple scales and its application in fire management. Ecol. Model..

[28] Finney MA (2011). A method for ensemble wildland fire simulation. Environ. Model. Assess..

[29] Davis C (2008). Prediction of landfalling hurricanes with the advanced hurricane WRF model. Mon. Weather Rev..

[30] Chen X, Zhan FB (2014). Agent-Based Modeling and Simulation.

[31] Yin W, Murray-Tuite P, Ukkusuri SV, Gladwin H (2014). An agent-based modeling system for travel demand simulation for hurricane evacuation. Transp. Res. Part C Emerg. Technol..

[32] Rahmandad H, Sterman J (2008). Heterogeneity and network structure in the dynamics of diffusion: Comparing agent-based and differential equation models. Manag. Sci..

[33] Bauch CT, Sigdel R, Pharaon J, Anand M (2016). Early warning signals of regime shifts in coupled human–environment systems. Proc. Natl. Acad. Sci..

[34] Innes C, Anand M, Bauch CT (2013). The impact of human–environment interactions on the stability of forest-grassland mosaic ecosystems. Sci. Rep..

[35] Russo L, Spiliotis K, Giannino F, Mazzoleni S, Siettos C (2019). Bautin bifurcations in a forest-grassland ecosystem with human–environment interactions. Sci. Rep..

[36] Staver AC, Levin SA (2012). Integrating theoretical climate and fire effects on savanna and forest systems. Am. Nat..

[37] Schertzer E, Staver A, Levin SA (2015). Implications of the spatial dynamics of fire spread for the bistability of savanna and forest. J. Math. Biol..

[38] Fiddaman TS (2002). Exploring policy options with a behavioral climate–economy model. Syst. Dyn. Rev. J. Syst. Dyn. Soc..

[39] Sterman J (2015). World climate: A role-play simulation of climate negotiations. Simul. Gaming.

[40] Deegan MA (2007). Exploring US Flood Mitigation Policies: A Feedback View of System Behavior.

[41] Richardson GP (2011). Reflections on the foundations of system dynamics. Syst. Dyn. Rev..

[42] Ghaffarzadegan N, Lyneis J, Richardson GP (2011). How small system dynamics models can help the public policy process. Syst. Dyn. Rev..

[43] Ghaffarzadegan N, Larson RC (2018). SD meets OR: A new synergy to address policy problems. Syst. Dyn. Rev..

[44] Sterman J (2000). Business Dynamics.

[45] McGee TK, McFarlane BL, Varghese J (2009). An examination of the influence of hazard experience on wildfire risk perceptions and adoption of mitigation measures. Soc. Nat. Resour..

[46] Martin WE, Martin IM, Kent B (2009). The role of risk perceptions in the risk mitigation process: The case of wildfire in high risk communities. J. Environ. Manag..

[47] Champ PA, Brenkert-Smith H (2016). Is seeing believing? Perceptions of wildfire risk over time. Risk Anal..

[48] McCaffrey S (2004). Thinking of wildfire as a natural hazard. Soc. Nat. Resour..

[49] Massada AB, Syphard AD, Stewart SI, Radeloff VC (2012). Wildfire ignition-distribution modelling: a comparative study in the Huron-Manistee National Forest, Michigan, USA. Int. J. Wildland Fire.

[50] Mancini LD (2018). Are wildfires knocking on the built-up areas door?. Forests.

[51] Gralewicz NJ, Nelson TA, Wulder MA (2011). Spatial and temporal patterns of wildfire ignitions in Canada from 1980 to 2006. Int. J. Wildland Fire.

[52] Meldrum JR (2019). Interactions between resident risk perceptions and wildfire risk mitigation: evidence from simultaneous equations modeling. Fire.

[53] Mochizuki J, Mechler R, Hochrainer-Stigler S, Keating A, Williges K (2014). Revisiting the ‘disaster and development’ debate–Toward a broader understanding of macroeconomic risk and resilience. Clim. Risk Manag..

[54] Botzen WW, Deschenes O, Sanders M (2019). The economic impacts of natural disasters: A review of models and empirical studies. Rev. Environ. Econ. Policy.

[55] Corona, P. *et al.* Integrated forest management to prevent wildfires under Mediterranean environments. (2015).

