# Supplementary material for: A cyclical wildfire pattern as the outcome of a coupled human natural system
Source: Sci Rep. 2022 Mar 28;12:5280. doi: 10.1038/s41598-022-08730-y (PMC8960864; doi:10.1038/s41598-022-08730-y)
Supplement: Supplementary file 1 — Supplementary Information. [file 41598_2022_8730_MOESM1_ESM.docx]

**Appendix 1. Model formulation**

Table A1 shows the full set of equations for the simulated model. In any program, these equations can be used to build a model. Nevertheless, these are used primarily to build a model in the Vensim DSS program. The Vensim DSS platform is an upgraded version of the Vensim PLE program that is available free for academic purposes. The DSS edition has a handful more options including optimization and sensitivity analysis. The model can therefore be developed using the Vensim PLE version with the following equations. The time stage for all simulations is 0.0078125 years. The model is run for 20 years but can be run for any other period.

**Table A1.** Complete set of model's equations, definition of parameters and units

| Parameter name | Parameter equation and definition and units of its parameters |
| --- | --- |
| 1. Vulnerable property | $V=\int_{0}^{t} (\left( \theta V \right)e^{-k\bar{B}}-\rho V)dt+I_{V}$ |
| 1. Indicated human ignition | $i_{H}=V\times E_{bt}\times h_{m}$ |
| 1. Human ignition | $I_{H}=$Third order delay of $i_{H}$;  delay duration = $\delta_{2}$; $I_{H}\left( t=0 \right)=0.3$ |
| 1. Burning rate | $B=\gamma_{F}F+\gamma_{S}S$ |
| 1. Strong vegetation | $S=\int_{0}^{t} \left( \frac{F}{\tau_{1}}-\left( \alpha+\gamma_{S} \right)S \right)dt+ I_{s}$ |
| 1. Flammable vegetation | $F=\int_{0}^{t} (\frac{E}{\tau_{2}}+\alpha S-(\frac{1}{\tau_{1}}+\gamma_{F})F)dt+ I_{F}$ |
| 1. Empty area | $E=\int_{0}^{t} \left( -\frac{dF}{dt}-\frac{dS}{dt} \right)dt+ I_{E}$ |
| 1. Vulnerable property development | $V_{i}=$ $E_{bt}\times\vartheta$ $\times V$ |
| 1. VP depreciation | $V_{o}=$ $V$ $\times E_{bv}$ |
| 1. Vegetation regrowing | $V_{r}= \frac{E}{\tau_{1}}$ |
| 1. Turning S to F | $T_{sf}=\sigma\times B\times S$ |
| 1. Total ignition | $T_{i}=$ $I_{H}$+ $I_{N}$ |
| 1. Strong vegetation burning | $S_{br}=$ $\rho$ $\times F_{p}\times S$ |
| 1. Flammable vegetation burning | $\gamma_{F}= f_{r}\times F\times T_{i}$ |
| 1. Fire propagation | $F_{p}=$ $0.8*{(1+e^{-5*(\frac{\gamma_{F}F}{n}-1)})}^{-1}$ |
| 1. Effect of BR on VP dep | $E_{bv}=$ $B$ $\times\mu$ |
| 1. Effect of BR on taking risk | $E_{bt}=$ max($\varpi$ +$\psi$ $\times\bar{B}$ , 0) |
| 1. Developing S | $D_{s}=\frac{F}{\tau_{2}}$ |
| 1. Burning effect on vulnerability | $B_{ev}=$ $B\times\sigma$ |
| 1. Fire risk perception | $\bar{B}=$ $Smooth(B$, $\delta_{1}$) |
| 1. Fractional development | $\theta=0.4$ |
| 1. VP deterioration effect | $\mu=0.2$ |
| 1. Risk intercept | $\varpi=1$ |
| 1. Risk multiplier | $\psi=-8$ |
| 1. Fractional burning rate per ignition | $f_{r}=0.08$ |
| 1. Average s burning | $\rho=6$ |
| 1. Time to change behavior | $\delta_{2}=2$ |
| 1. Human ignition multiplier | $h_{m}=5$ |
| **29.** Time to grow vegetation | $\tau_{1}=$ 2 |
| **30.** Time to develop S | $\tau_{2}=$ 10 |
| **31.** Time to perceive | $\delta_{1}=$ 0.5 |
| **32.** Initial strong vegetation | $S\left( t=0 \right)=$ 0.5 |
| **33.** Initial flammable vegetation | $F\left( t=0 \right) =$ 0.4 |
| **34.** Initial empty area | $E\left( t=0 \right)=$ 0.1 |
| **35.** Fire propagation | $\gamma_{S}= 0.8\times{(1+e^{-5*(\frac{\gamma_{F}F}{n}-1)})}^{-1}$ |
| **36.** Normal burning | $n=$ 0.1 |
| **37.** Natural ignition | $I_{N}=$0.5 |
| **38.** Initial human ignition | $I_{H}(t=0)=$ 0.3 |
| **39.** Initial vulnerable property | $V(t=0)$ = 0.4 |
| **40.** BR multiplier | $\sigma$ = 0.05 |

**Appendix 2. Sensitivity Analysis**

We use Monte-Carlo Analysis to determine the model's robustness to parameter variations. The emphasis is on parameters that can take on values other than those assumed in the model. These parameters are risk intercept, risk multiplier, time to perceive risk, time to change behavior, Fractional burning rate per ignition, average s burning, initial flammable vegetation, initial strong vegetation, human ignition multiplier, and initial vulnerable property. We checked the sensitivity of the burning rate compared to these variables. For most of these variables (except initial flammable vegetation and initial strong vegetation), we changed the corresponding variable up to double its base run value. We modify the values for initial strong vegetation and initial flammable vegetation between zero and their base run values, as they indicate the fraction of the forest that each occupies. Each sensitivity test is the outcome of 2000 simulation runs using a uniformly distributed random distribution of the parameters within the defined intervals. As illustrated in Figure A1, the results are qualitatively robust, and their variability is within reasonable limits.

Figure A1: sensitivity analysis for change in parameters.

| Risk intercept | Risk multiplier |
| --- | --- |
| 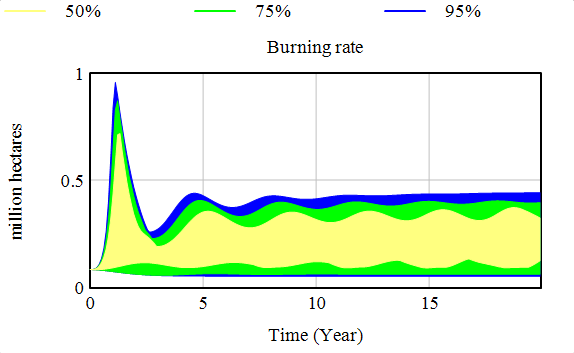 | 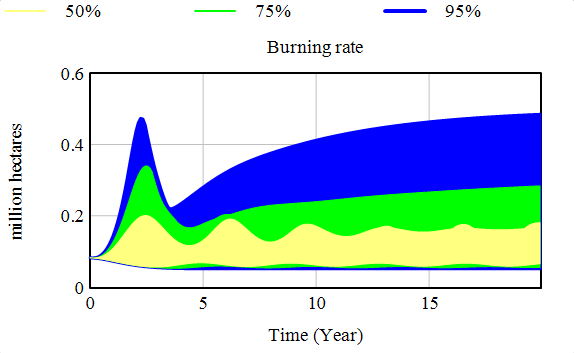 |
| Time to perceive risk | Time to change behavior |
| 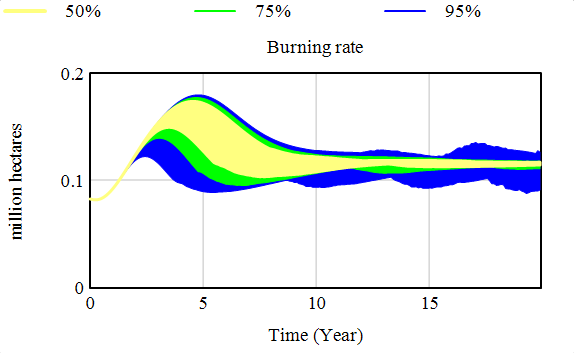 | 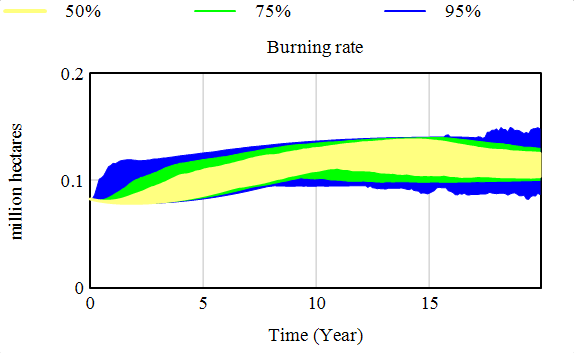 |

| Fractional burning rate per ignition | Average s burning |
| --- | --- |
| 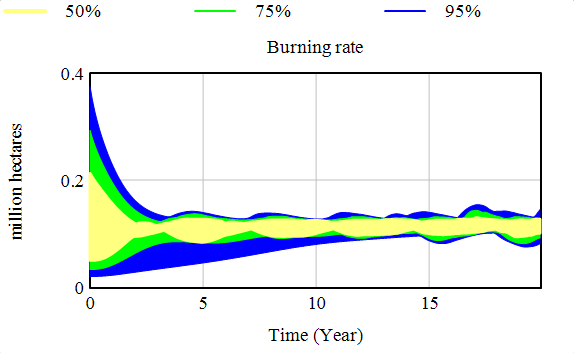 | 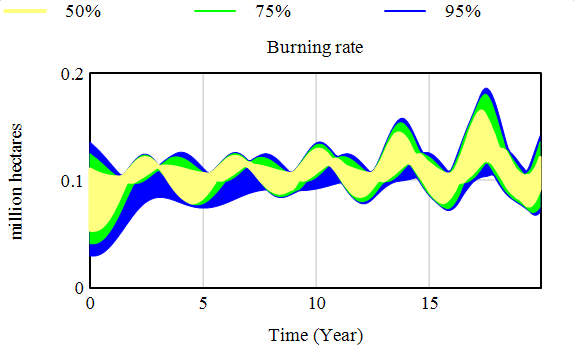 |
| Initial falammable vegetation | Initial strong vegetation |
| 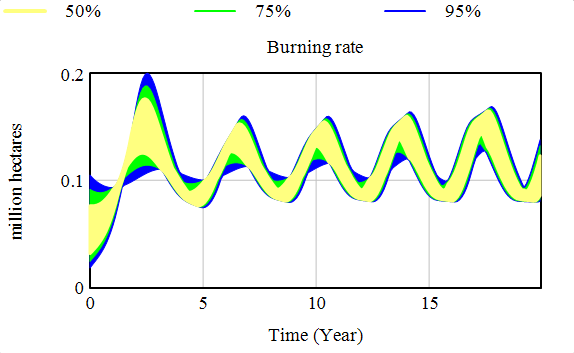 | 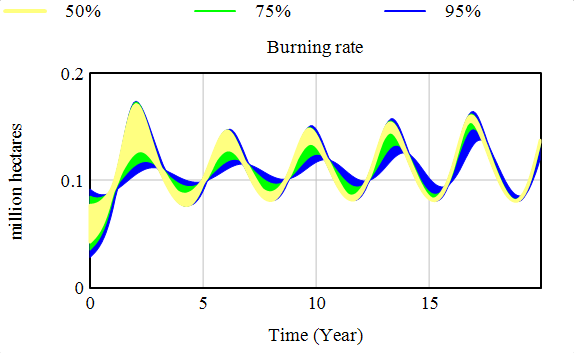 |
| Human ignition multiplier | Initial vulnerable property |
| 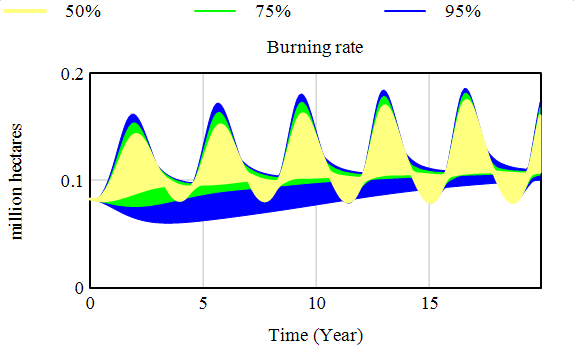 | 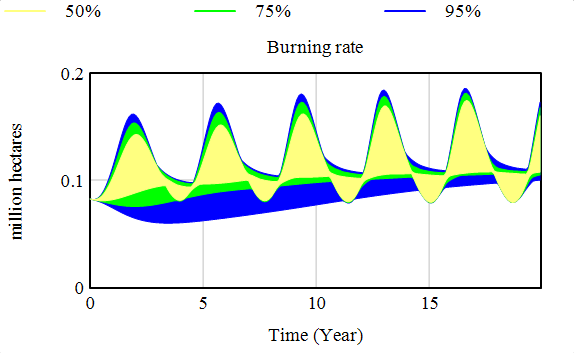 |

**Appendix 3. Policy Analysis Implementation**

Table A2 includes a list of equation adjustments necessary to execute each of the four suggested policies to ease the policy analysis. The equations are sequentially numbered per Table A1. Because each policy is implemented at time 5, we utilize the pulse function, zero before and one after this time.

Table A2. Complete set of adjustments in equations for each policy

| Policy | Equation number | New Equation |
| --- | --- | --- |
| P1: Limit vulnerable property development. | 8 | $E_{bt}\times\vartheta\times V\times\left( 1-PULSE\left( 5,15 \right) \right)+0.01\times V\times PULSE(5,15)$ |
| P2: Prescribed burning. | 6 | $\int_{0}^{t} (\frac{E}{\tau_{2}}+\alpha S-\left( \frac{1}{\tau_{1}}+\gamma_{F} \right)F-\omega F\times PULSE\left( 5,15 \right))dt+ I_{F}$ |
| P3: Effective firefighting. | 35 | $(0.8-0.72\times PULSE(5,15)\times{(1+e^{-5*(\frac{\gamma_{F}F}{n}-1)})}^{-1}$ |
| P4: Clear cutting. | 5 | $\int_{0}^{t} \left( \frac{F}{\tau_{1}}-\left( \alpha+\gamma_{S} \right)S-\vartheta S\times PULSE\left( 5,15 \right) \right)dt+ I_{s}$ |

**Appendix 4. Increasing simulation timeline for base run simulation**

Figure A2: The burning rate for the base run for 100 years timespan
